# Supplementary figures and images for: Screening microbial inhibitors of Pseudogymnoascus destructans in Northern China
Source: Microbiol Spectr. 2025 Oct 23;13(12):e01241-25. doi: 10.1128/spectrum.01241-25 (PMC12671219; doi:10.1128/spectrum.01241-25)

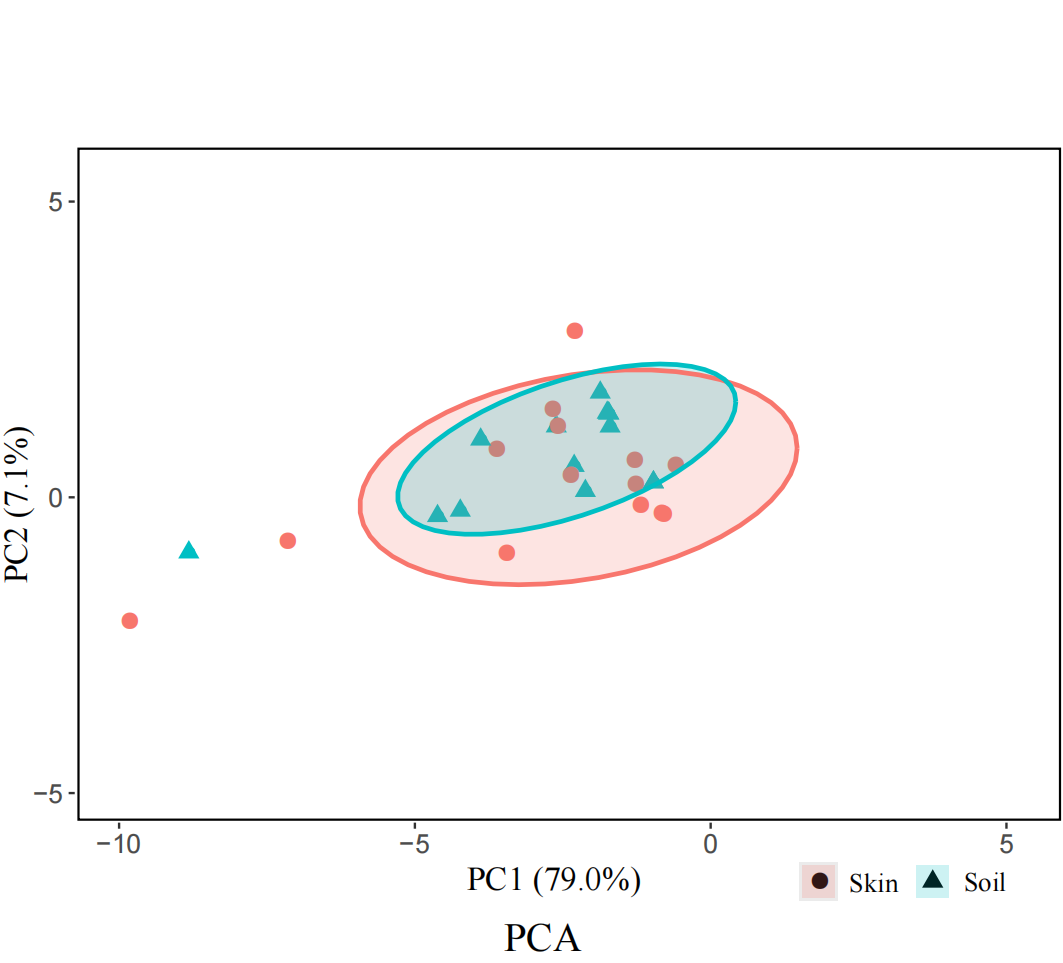

Supplement: Figure S1 — PCA analysis of biosynthetic gene clusters of antagonistic strains against P. destructans from bat skin and soil samples. [file spectrum.01241-25-s0001.tif]

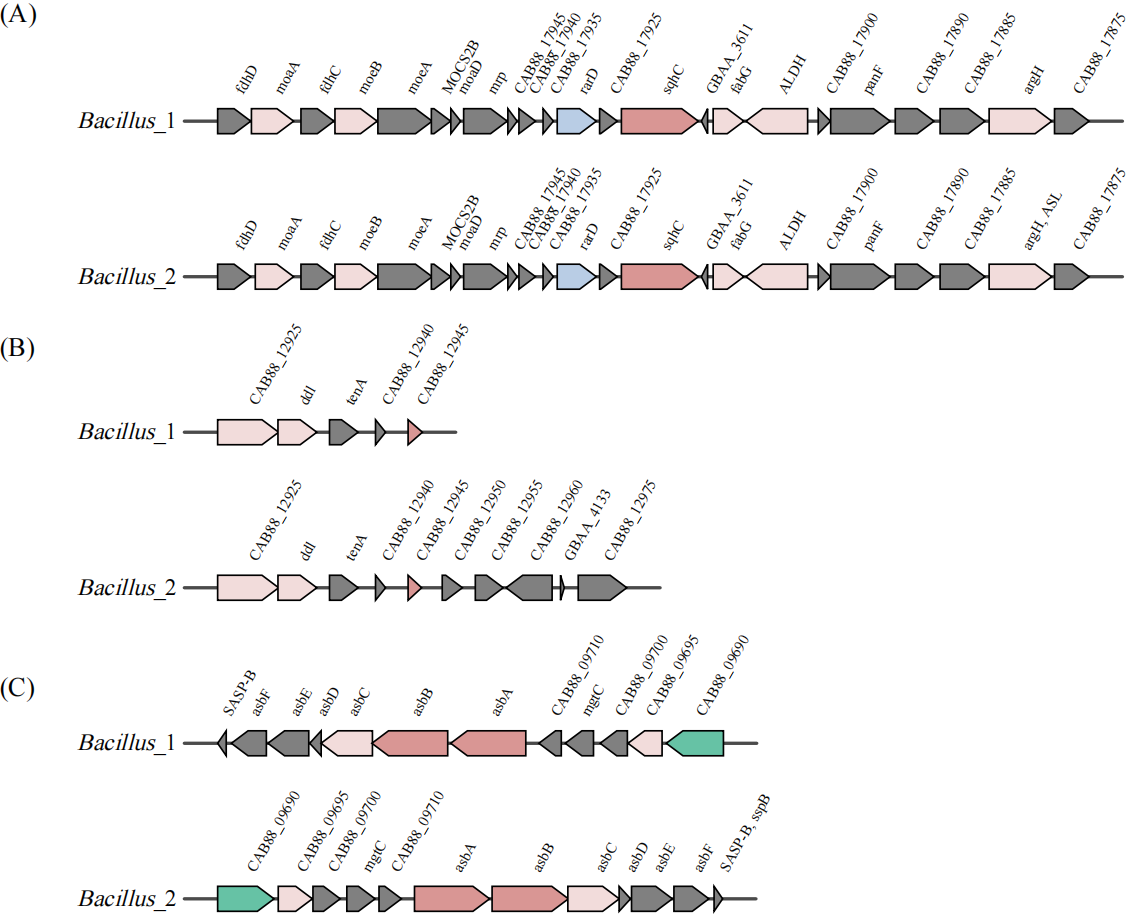

Supplement: Figure S2 — Comparative analysis of biosynthetic gene clusters in the Bacillus. [file spectrum.01241-25-s0002.tif]
