# Supplementary material for: Screening microbial inhibitors of Pseudogymnoascus destructans in Northern China
Source: Microbiol Spectr. 2025 Oct 23;13(12):e01241-25. doi: 10.1128/spectrum.01241-25 (PMC12671219; doi:10.1128/spectrum.01241-25)
Supplement: Tables S1 to S5 — Support information for screening microbial inhibitors of Pseudogymnoascus destructans in northern China. [file spectrum.01241-25-s0003.docx]

**Supplementary material**

Table S1 Types and relative abundances (>1%) of volatile organic compounds produced by antagonistic strains against *P. destructans* from bat skin and soil.

| Strains | VOCs | CAS | Relative abundances |
| --- | --- | --- | --- |
| *Acinetobacter*_1 | [1R,5R,(-)]-2-Isopropylidene-N,N,5-Trimethylcyclopentanemethanamine | 17943-83-8 | 1.07% |
| *Acinetobacter*_1 | Dimethyl sulfide | 75-18-3 | 1.43% |
| *Acinetobacter_*1 | Pyrazine, methyl- | 109-08-0 | 1.11% |
| *Acinetobacter_*1 | Benzocyclobutene | 694-87-1 | 2.30% |
| *Acinetobacter_*1 | Pyrazine, 2,5-dimethyl- | 123-32-0 | 1.81% |
| *Acinetobacter_*1 | (+)-α-Pinene | 7785-70-8 | 2.27% |
| *Acinetobacter_*1 | 1-Undecene | 821-95-4 | 10.71% |
| *Acinetobacter_*1 | 2-Undecanone | 112-12-9 | 1.75% |
| *Acinetobacter_*1 | 2-Tridecanone | 593-08-8 | 4.69% |
| *Acinetobacter_*1 | Cyclodecasiloxane, eicosamethyl- | 18772-36-6 | 1.36% |
| *Acinetobacter_*10 | 2-Butanone | 78-93-3 | 4.40% |
| *Acinetobacter_*10 | Pyrazine, methyl- | 109-08-0 | 1.30% |
| *Acinetobacter_*10 | Pyrazine, 2,5-dimethyl- | 123-32-0 | 3.00% |
| *Acinetobacter_*10 | 2-Hydroxy-3-methoxybenzaldehyde, tert-butyldimethylsilyl ether | 1000352-86-2 | 1.20% |
| *Acinetobacter_*10 | 1-Undecene | 821-95-4 | 1.20% |
| *Acinetobacter_*10 | 2-Phenylethanol | 60-12-8 | 10.20% |
| *Acinetobacter_*10 | Palmitic acid | 57-10-3 | 2.10% |
| *Acinetobacter_*11 | 1-Undecene | 821-95-4 | 1.70% |
| *Acinetobacter_*11 | 2-Phenylethanol | 60-12-8 | 14.20% |
| *Acinetobacter_*11 | (Z)6-Pentadecen-1-ol | 68797-95-5 | 1.70% |
| *Acinetobacter_*11 | n-Nonadecanol-1 | 1454-84-8 | 3.70% |
| *Acinetobacter_*11 | E-15-Heptadecenal | 1000130-97-9 | 1.40% |
| *Acinetobacter_*12 | Pyrazine, 2,5-dimethyl- | 123-32-0 | 3.60% |
| *Acinetobacter_*12 | Decane, 3-methyl- | 13151-34-3 | 1.10% |
| *Acinetobacter_*12 | Benzaldehyde | 100-52-7 | 1.10% |
| *Acinetobacter_*12 | Bicyclo[3.1.0]hexan-3-one, 4-methyl-1-(1-methylethyl)- | 1125-12-8 | 10.50% |
| *Acinetobacter_*12 | Undecane, 3,8-dimethyl- | 17301-30-3 | 2.10% |
| *Acinetobacter_*12 | Cyclohexanol, 2,3-dimethyl- | 1502-24-5 | 2.70% |
| *Acinetobacter_*12 | Decane, 3,8-dimethyl- | 17312-55-9 | 2.10% |
| *Acinetobacter_*12 | Undecane, 4-methyl- | 2980-69-0 | 1.40% |
| *Acinetobacter_*12 | 2-Methylundecane | 7045-71-8 | 2.80% |
| *Acinetobacter_*12 | Undecane, 3-methyl- | 1002-43-3 | 3.60% |
| *Acinetobacter_*12 | Isooctane, (ethenyloxy)- | 37769-62-3 | 2.50% |
| *Acinetobacter_*12 | Hexasiloxane, tetradecamethyl- | 107-52-8 | 2.30% |
| *Acinetobacter_*12 | Menthol, 1'-(butyn-3-one-1-yl)-, (1R,2S,5R)- | 1000156-95-4 | 1.10% |
| *Acinetobacter_*12 | Heptasiloxane, hexadecamethyl- | 541-01-5 | 1.60% |
| *Acinetobacter_*16 | 1-Undecene | 821-95-4 | 6.94% |
| *Acinetobacter_*16 | 2-Nonanone | 821-55-6 | 8.80% |
| *Acinetobacter_*16 | 2-Undecanone | 112-12-9 | 10.84% |
| *Acinetobacter_*16 | 11-Dodecen-2-one | 5009-33-6 | 1.78% |
| *Acinetobacter_*16 | (Z)-Dec-3-en-1-yl acetate | 81634-99-3 | 4.44% |
| *Acinetobacter_*16 | 2-Tridecanone | 593-08-8 | 3.58% |
| *Acinetobacter_*16 | Cyclopentadecanone | 502-72-7 | 1.86% |
| *Acinetobacter_*16 | 3,4'-Diisopropyl-1,1'-biphenyl | 61434-46-6 | 1.82% |
| *Acinetobacter_*17 | Pyrazine, 2,5-dimethyl- | 123-32-0 | 1.27% |
| *Acinetobacter_*17 | 1-Undecene | 821-95-4 | 4.13% |
| *Acinetobacter_*17 | 2-Nonanone | 821-55-6 | 14.59% |
| *Acinetobacter_*17 | 2-Undecanone | 112-12-9 | 15.15% |
| *Acinetobacter_*17 | 2-Dodecanone | 6175-49-1 | 1.21% |
| *Acinetobacter_*17 | 11-Dodecen-2-one | 5009-33-6 | 3.35% |
| *Acinetobacter_*17 | 2-Tridecanone | 593-08-8 | 2.55% |
| *Acinetobacter_*17 | (Z)-Dec-3-en-1-yl acetate | 81634-99-3 | 1.30% |
| *Acinetobacter_*18 | 2-Nonanone | 821-55-6 | 5.11% |
| *Acinetobacter_*18 | 2-Undecanone | 112-12-9 | 4.85% |
| *Acinetobacter_*18 | Oxirane, dodecyl- | 3234-28-4 | 11.15% |
| *Acinetobacter_*18 | (Z)-Dec-3-en-1-yl acetate | 81634-99-3 | 8.79% |
| *Acinetobacter_*18 | 2-Tridecanone | 593-08-8 | 2.07% |
| *Acinetobacter_*18 | Ethanone, 1-cyclododecyl- | 28925-00-0 | 2.09% |
| *Acinetobacter_*18 | Cyclopentadecanone | 502-72-7 | 3.23% |
| *Acinetobacter_*19 | Pyrazine, methyl- | 109-08-0 | 4.20% |
| *Acinetobacter_*19 | Pyrazine, 2,5-dimethyl- | 123-32-0 | 2.00% |
| *Acinetobacter_*19 | Benzaldehyde | 100-52-7 | 25.50% |
| *Acinetobacter_*19 | (1R)-(−)-Fenchone | 7787-20-4 | 1.10% |
| *Acinetobacter_*19 | Bicyclo[3.1.0]hexan-3-one, 4-methyl-1-(1-methylethyl)- | 1125-12-8 | 6.10% |
| *Acinetobacter_*19 | Menthol, 1'-(butyn-3-one-1-yl)-, (1R,2S,5R)- | 1000156-95-4 | 1.40% |
| *Acinetobacter_*19 | 2-Tridecanone | 593-08-8 | 2.40% |
| *Acinetobacter_*19 | 2-Tetradecanone | 2345-27-9 | 3.40% |
| *Acinetobacter_*19 | Cyclohexanone, 2-methyl-2-(3-methyl-2-oxobutyl) | 871331-14-5 | 3.30% |
| *Acinetobacter_*19 | (E/Z)-Squalene | 7683-64-9 | 2.40% |
| *Acinetobacter_*2 | Borane-methyl sulfide complex | 13292-87-0 | 1.98% |
| *Acinetobacter_*2 | Benzene, 1-methyl-3-(1-methylethyl)- | 535-77-3 | 1.13% |
| *Acinetobacter_*2 | 1-Undecene | 821-95-4 | 1.40% |
| *Acinetobacter_*2 | 2-Nonanone | 821-55-6 | 19.20% |
| *Acinetobacter_*2 | Bicyclo[3.1.0]hexan-3-one, 4-methyl-1-(1-methylethyl)- | 1125-12-8 | 3.03% |
| *Acinetobacter_*2 | 2-Undecanone | 112-12-9 | 2.82% |
| *Acinetobacter_*2 | 2-Tridecanone | 593-08-8 | 4.48% |
| *Acinetobacter_*2 | Heneicosane | 629-94-7 | 1.12% |
| *Acinetobacter_*2 | Cyclodecasiloxane, eicosamethyl- | 18772-36-6 | 1.24% |
| *Acinetobacter_*4 | Tricyclo[3.3.0.0(2,8)]octan-3-one, 5,8-dimethyl- | 109915-32-4 | 3.44% |
| *Acinetobacter_*4 | trans-3-Caren-2-ol | 1000151-75-4 | 1.16% |
| *Acinetobacter_*4 | Octadecanoic acid, ethenyl ester | 111-63-7 | 1.81% |
| *Acinetobacter_*4 | 2-Undecanone | 112-12-9 | 2.91% |
| *Acinetobacter_*4 | 1,3-Dioxane, 4-(hexadecyloxy)-2-pentadecyl- | 56599-40-7 | 1.79% |
| *Acinetobacter_*4 | 2-Tridecanone | 593-08-8 | 8.69% |
| *Acinetobacter_*4 | Cyclodecasiloxane, eicosamethyl- | 18772-36-6 | 2.37% |
| *Acinetobacter_*6 | Oxime-, methoxy-phenyl-_ | 3235-04-9 | 1.20% |
| *Acinetobacter_*6 | Fluoren-9-ol, 3,6-dimethoxy-9-(2-phenylethynyl)- | 1000217-31-2 | 1.10% |
| *Acinetobacter_*6 | 2-Undecanone | 112-12-9 | 1.70% |
| *Acinetobacter_*6 | 2-Dodecanone | 6175-49-1 | 2.20% |
| *Acinetobacter_*6 | 1-Eicosanol | 629-96-9 | 14.60% |
| *Acinetobacter_*6 | Menthol, 1'-(butyn-3-one-1-yl)-, (1R,2S,5R)- | 1000156-95-4 | 1.70% |
| *Acinetobacter_*6 | Ethanone, 1-cyclododecyl- | 28925-00-0 | 1.20% |
| *Acinetobacter_*9 | 2-Phenylethanol | 60-12-8 | 15.50% |
| *Acinetobacter_*9 | 2-Undecanone | 112-12-9 | 1.70% |
| *Acinetobacter_*9 | Octadecyl Vinyl Ether | 930-02-9 | 1.30% |
| *Pantoea_*1 | 1-Undecene | 821-95-4 | 6.63% |
| *Pantoea_*1 | Bicyclo[3.1.0]hexan-3-one, 4-methyl-1-(1-methylethyl)- | 1125-12-8 | 1.12% |
| *Pseudomonas_*1 | Benzene, 1-methyl-3-(1-methylethyl)- | 535-77-3 | 1.62% |
| *Pseudomonas_*1 | 2-Nonanone | 821-55-6 | 1.92% |
| *Pseudomonas_*1 | Bicyclo[3.1.0]hexan-3-one, 4-methyl-1-(1-methylethyl)- | 1125-12-8 | 2.47% |
| *Pseudomonas_*1 | 1,3-Dioxane, 5-(hexadecyloxy)-2-pentadecyl-, cis- | 34298-21-0 | 2.10% |
| *Pseudomonas_*1 | 1-Heptadecene | 6765-39-5 | 1.42% |
| *Pseudomonas_*1 | Cyclodecasiloxane, eicosamethyl- | 18772-36-6 | 1.36% |
| *Pseudomonas_*2 | Thiocyanic acid, methyl ester | 556-64-9 | 3.00% |
| *Pseudomonas_*2 | 1-Undecene | 821-95-4 | 15.90% |
| *Pseudomonas_*2 | 2-Nonanone | 821-55-6 | 2.70% |
| *Pseudomonas_*2 | Palmitic acid | 57-10-3 | 1.70% |
| *Pseudomonas_*3 | 1-Undecene | 821-95-4 | 18.30% |
| *Pseudomonas_*3 | 2-Nonanone | 821-55-6 | 3.70% |
| *Pseudomonas_*3 | n-Nonadecanol-1 | 1454-84-8 | 1.60% |
| *Pseudomonas_*3 | Hexadecanoic acid, butyl ester | 111-06-8 | 1.80% |
| *Pseudomonas_*4 | 1-Undecene | 821-95-4 | 10.40% |
| *Pseudomonas_*4 | 1-Nonadecene | 18435-45-5 | 3.90% |
| *Pseudomonas_*4 | Cycloeicosane | 296-56-0 | 1.50% |
| *Serratia_*1 | Benzene, 1-methyl-3-(1-methylethyl)- | 535-77-3 | 1.13% |
| *Serratia_*1 | Bicyclo[3.1.0]hexan-3-one, 4-methyl-1-(1-methylethyl)- | 1125-12-8 | 2.30% |
| *Serratia_*1 | 2-Undecanone | 112-12-9 | 10.64% |
| *Serratia_*1 | 2-Dodecanone | 6175-49-1 | 8.25% |
| *Serratia_*1 | 2-Tridecanone | 593-08-8 | 25.37% |
| *Serratia_*1 | 2-Tetradecanone | 2345-27-9 | 1.49% |
| *Serratia_*1 | Cyclodecasiloxane, eicosamethyl- | 18772-36-6 | 1.36% |
| *Stenotrophomonas_*1 | D-Limonene | 138-86-3 | 2.70% |
| *Stenotrophomonas_*1 | Isopar G | 6975-98-0 | 1.20% |
| *Stenotrophomonas_*1 | Undecane, 4,7-dimethyl- | 17301-32-5 | 1.20% |
| *Stenotrophomonas_*1 | 2-Methylundecane | 7045-71-8 | 5.20% |
| *Stenotrophomonas_*1 | Undecane, 3-methyl- | 1002-43-3 | 5.10% |
| *Stenotrophomonas_*1 | Undecane, 5-methyl- | 1632-70-8 | 2.60% |
| *Stenotrophomonas_*1 | Undecane, 4-methyl- | 2980-69-0 | 2.00% |
| *Stenotrophomonas_*1 | Undecane, 2,5-dimethyl- | 17301-22-3 | 1.10% |
| *Stenotrophomonas_*1 | 2,2-DIMETHYL-N-PHENETHYL-PROPIONAMIDE | 62056-54-6 | 1.90% |
| *Stenotrophomonas_*1 | 5,9,13-Pentadecatrien-2-one, 6,10,14-trimethyl-, (E,E)- | 1117-52-8 | 1.10% |

Table S2 The determination of the non-protein and protein components' activity and the molecular weight range of active compounds in the fermentation broth of antagonistic strains.

| Strains | Protein | Non-protein | Within a 3k dialysis bag | Outside a 3k dialysis bag | Within a 1k dialysis bag | Outside a 1k dialysis bag |
| --- | --- | --- | --- | --- | --- | --- |
| *Acinetobacter_*1 | 0 | 36.20% | 0 | 27.25% | 0 | 37.99% |
| *Acinetobacter_*2 | 0 | 94.47% | 0 | 37.88% | 0 | 43.99% |
| *Mammaliicoccus_*1 | 23.15% | 61.35% | 0 | 70.00% | 0 | 59.72% |
| *Acinetobacter_*3 | 0 | 48.63% | 0 | 72.41% | 0 | 44.59% |
| *Staphylococcus_*1 | 0 | 73.10% | 0 | 43.10% | 0 | 48.36% |
| *Bacillus_*1 | 0 | 45.86% | 0 | 58.21% | 0 | 51.63% |
| *Acinetobacter_*4 | 0 | 57.85% | 0 | 42.28% | 0 | 47.23% |
| *Pantoea_*1 | 0 | 91.39% | 0 | 59.30% | 0 | 58.40% |
| *Serratia_*1 | 0 | 41.15% | 0 | 76.65% | 0 | 86.00% |
| *Bacillus_*2 | 0 | 84.62% | 0 | 36.83% | 0 | 63.69% |
| *Acinetobacter_*5 | 0 | 32.71% | 0 | 49.93% | 0 | 48.11% |
| *Acinetobacter_*6 | 0 | 35.96% | 0 | 31.26% | 0 | 44.63% |
| *Acinetobacter_*7 | 0 | 89.60% | 0 | 50.67% | 0 | 44.43% |
| *Acinetobacter_*8 | 0 | 80.49% | 0 | 46.86% | 0 | 47.75% |
| *Acinetobacter_*12 | 0 | 45.23% | 0 | 39.36% | 0 | 45.62% |
| *Acinetobacter_*13 | 0 | 78.43% | 0 | 50.24% | 0 | 82.97% |
| *Acinetobacter_*15 | 0 | 81.74% | 0 | 43.50% | 0 | 45.13% |

Table S3 Biosynthetic gene clusters of antagonistic strains.

| Sample ID | Clusters | Clusters_number | Gene_number |
| --- | --- | --- | --- |
| *Acinetobacter_*1 | NRPS, hserlactone | 1 | 50 |
| *Acinetobacter_*1 | NRPS | 1 | 32 |
| *Acinetobacter_*1 | arylpolyene | 1 | 24 |
| *Acinetobacter_*1 | bacteriocin | 1 | 14 |
| *Acinetobacter_*2 | arylpolyene | 2 | 70 |
| *Acinetobacter_*2 | bacteriocin | 1 | 13 |
| *Acinetobacter_*2 | betalactone | 2 | 40 |
| *Acinetobacter_*2 | NAGGN | 1 | 7 |
| *Acinetobacter_*2 | siderophore | 2 | 20 |
| *Acinetobacter_*2 | NRPS | 1 | 47 |
| *Acinetobacter_*2 | NRPS, hserlactone | 1 | 51 |
| *Mammaliicoccus_*1 | terpene | 1 | 13 |
| *Mammaliicoccus_*1 | siderophore | 2 | 24 |
| *Mammaliicoccus_*1 | T3PKS | 2 | 76 |
| *Staphylococcus_*1 | NRPS | 1 | 31 |
| *Staphylococcus_*1 | T3PKS | 1 | 34 |
| *Staphylococcus_*1 | terpene | 1 | 31 |
| *Staphylococcus_*1 | siderophore | 1 | 12 |
| *Bacillus_*1 | terpene | 1 | 23 |
| *Bacillus_*1 | LAP, bacteriocin | 1 | 24 |
| *Bacillus_*1 | siderophore | 1 | 12 |
| *Bacillus_*1 | NRPS | 3 | 41 |
| *Bacillus_*1 | CDPS | 1 | 7 |
| *Bacillus_*1 | bacteriocin | 1 | 5 |
| *Bacillus_*1 | lanthipeptide | 2 | 40 |
| *Bacillus_*1 | NRPS-like | 1 | 37 |
| *Bacillus_*1 | lassopeptide | 1 | 1 |
| *Bacillus_*1 | betalactone | 1 | 25 |
| *Acinetobacter_*4 | NRPS | 1 | 48 |
| *Acinetobacter_*4 | LAP | 1 | 20 |
| *Pantoea_*1 | hserlactone | 1 | 21 |
| *Pantoea_*1 | thiopeptide | 1 | 16 |
| *Pantoea_*1 | arylpolyene, hserlactone | 1 | 68 |
| *Pantoea_*1 | siderophore | 1 | 9 |
| *Pantoea_*1 | terpene | 1 | 29 |
| *Pantoea_*1 | NRPS | 1 | 53 |
| *Serratia_*1 | CDPS | 1 | 17 |
| *Serratia_*1 | hserlactone | 1 | 63 |
| *Serratia_*1 | butyrolactone, ectoine | 1 | 18 |
| *Serratia_*1 | NRPS-like | 1 | 20 |
| *Serratia_*1 | NRPS, siderophore | 1 | 39 |
| *Serratia_*1 | thiopeptide | 1 | 19 |
| *Serratia_*1 | arylpolyene | 1 | 34 |
| *Serratia_*1 | bacteriocin | 1 | 12 |
| *Serratia_*1 | siderophore | 1 | 10 |
| *Serratia_*1 | NRPS | 3 | 77 |
| *Serratia_*1 | betalactone | 1 | 38 |
| *Bacillus_*2 | NRPS | 9 | 293 |
| *Bacillus_*2 | T3PKS | 1 | 34 |
| *Bacillus_*2 | NRPS-like | 1 | 35 |
| *Bacillus_*2 | terpene | 3 | 65 |
| *Bacillus_*2 | NRPS, T1PKS | 1 | 62 |
| *Bacillus_*2 | LAP, bacteriocin | 1 | 22 |
| *Bacillus_*2 | bacteriocin | 3 | 30 |
| *Bacillus_*2 | lanthipeptide | 2 | 48 |
| *Bacillus_*2 | betalactone | 1 | 27 |
| *Bacillus_*2 | siderophore | 2 | 24 |
| *Pseudomonas_*1 | LAP | 1 | 18 |
| *Pseudomonas_*1 | NRPS, terpene | 1 | 30 |
| *Pseudomonas_*1 | terpene | 1 | 17 |
| *Pseudomonas_*1 | betalactone | 2 | 45 |
| *Pseudomonas_*1 | betalactone | 1 | 40 |
| *Pseudomonas_*1 | bacteriocin | 3 | 36 |
| *Pseudomonas_*1 | NAGGN | 1 | 9 |
| *Pseudomonas_*1 | siderophore | 1 | 12 |
| *Pseudomonas_*1 | LAP, bacteriocin | 1 | 23 |
| *Pseudomonas_*1 | NRPS-like | 2 | 34 |
| *Pseudomonas_*1 | NRPS | 6 | 222 |
| *Acinetobacter_*5 | NRPS | 1 | 48 |
| *Acinetobacter_*6 | NRPS | 1 | 47 |
| *Acinetobacter_*6 | arylpolyene | 1 | 24 |
| *Acinetobacter_*7 | bacteriocin | 1 | 9 |
| *Acinetobacter_*7 | NRPS | 2 | 73 |
| *Acinetobacter_*7 | siderophore | 1 | 13 |
| *Acinetobacter_*7 | arylpolyene | 1 | 24 |
| *Acinetobacter_*8 | NRPS | 1 | 48 |
| *Acinetobacter_*8 | arylpolyene | 1 | 24 |
| *Acinetobacter_*9 | bacteriocin | 1 | 14 |
| *Acinetobacter_*9 | siderophore | 1 | 10 |
| *Acinetobacter_*9 | arylpolyene | 1 | 24 |
| *Acinetobacter_*9 | betalactone | 1 | 24 |
| *Acinetobacter_*9 | NRPS | 2 | 55 |
| *Acinetobacter_*9 | hserlactone | 1 | 11 |
| *Acinetobacter_*10 | betalactone | 1 | 24 |
| *Acinetobacter_*10 | bacteriocin | 1 | 14 |
| *Acinetobacter_*10 | arylpolyene | 1 | 24 |
| *Acinetobacter_*10 | siderophore | 1 | 10 |
| *Acinetobacter_*10 | NRPS, hserlactone | 1 | 51 |
| *Acinetobacter_*10 | NRPS | 1 | 32 |
| *Pseudomonas_*2 | NAGGN | 1 | 13 |
| *Pseudomonas_*2 | lassopeptide | 2 | 30 |
| *Pseudomonas_*2 | thiopeptide | 1 | 38 |
| *Pseudomonas_*2 | betalactone | 1 | 22 |
| *Pseudomonas_*2 | arylpolyene | 1 | 36 |
| *Pseudomonas_*2 | NRPS-like | 1 | 13 |
| *Pseudomonas_*2 | bacteriocin | 1 | 8 |
| *Pseudomonas_*2 | NRPS | 4 | 92 |
| *Acinetobacter_*11 | siderophore | 1 | 10 |
| *Acinetobacter_*11 | betalactone | 1 | 24 |
| *Acinetobacter_*11 | NRPS | 1 | 48 |
| *Acinetobacter_*11 | bacteriocin | 1 | 14 |
| *Acinetobacter_*11 | arylpolyene | 1 | 24 |
| *Acinetobacter_*11 | NRPS, hserlactone | 1 | 51 |
| *Pseudomonas_*3 | NRPS-like | 1 | 31 |
| *Pseudomonas_*3 | bacteriocin | 3 | 32 |
| *Pseudomonas_*3 | arylpolyene | 1 | 26 |
| *Pseudomonas_*3 | NRPS | 4 | 41 |
| *Pseudomonas_*3 | NAGGN | 1 | 10 |
| *Acinetobacter_*12 | bacteriocin | 1 | 14 |
| *Acinetobacter_*12 | arylpolyene | 1 | 24 |
| *Acinetobacter_*12 | betalactone | 1 | 24 |
| *Acinetobacter_*12 | siderophore | 1 | 10 |
| *Acinetobacter_*12 | NRPS, hserlactone | 1 | 51 |
| *Acinetobacter_*12 | NRPS | 1 | 48 |
| *Acinetobacter_*13 | arylpolyene | 1 | 23 |
| *Acinetobacter_*13 | siderophore | 1 | 10 |
| *Acinetobacter_*13 | bacteriocin | 1 | 14 |
| *Acinetobacter_*13 | NRPS,hserlactone | 1 | 51 |
| *Acinetobacter_*13 | NRPS | 1 | 32 |
| *Acinetobacter_*13 | betalactone | 1 | 24 |
| *Pseudomonas_*4 | betalactone | 2 | 37 |
| *Pseudomonas_*4 | arylpolyene | 1 | 8 |
| *Pseudomonas_*4 | NRPS | 8 | 111 |
| *Pseudomonas_*4 | hserlactone | 1 | 10 |
| *Pseudomonas_*4 | bacteriocin | 2 | 15 |
| *Pseudomonas_*4 | NRPS-like | 2 | 35 |
| *Pseudomonas_*4 | other | 1 | 8 |
| *Pseudomonas_*4 | terpene | 2 | 10 |
| *Pseudomonas_*4 | NRPS, T1PKS | 1 | 11 |
| *Pseudomonas_*4 | siderophore | 2 | 18 |
| *Acinetobacter_*15 | bacteriocin | 1 | 14 |
| *Acinetobacter_*15 | hserlactone | 2 | 11 |
| *Acinetobacter_*15 | NRPS | 1 | 43 |
| *Acinetobacter_*15 | betalactone | 1 | 24 |
| *Acinetobacter_*15 | arylpolyene | 1 | 24 |
| *Acinetobacter_*15 | siderophore | 1 | 10 |
| *Acinetobacter_*16 | arylpolyene | 1 | 8 |
| *Acinetobacter_*16 | bacteriocin | 1 | 10 |
| *Acinetobacter_*16 | siderophore | 1 | 8 |
| *Acinetobacter_*16 | betalactone | 1 | 19 |
| *Acinetobacter_*16 | hserlactone | 1 | 10 |
| *Acinetobacter_*16 | NRPS | 2 | 31 |
| *Acinetobacter_*17 | siderophore | 1 | 10 |
| *Acinetobacter_*17 | NRPS | 2 | 22 |
| *Acinetobacter_*17 | hserlactone | 2 | 21 |
| *Acinetobacter_*17 | betalactone | 1 | 24 |
| *Acinetobacter_*17 | arylpolyene | 1 | 24 |
| *Acinetobacter_*17 | bacteriocin | 1 | 12 |
| *Acinetobacter_*18 | bacteriocin | 1 | 8 |
| *Acinetobacter_*18 | betalactone | 1 | 13 |
| *Acinetobacter_*18 | siderophore | 1 | 8 |
| *Acinetobacter_*18 | NRPS | 2 | 31 |
| *Stenotrophomonas_*1 | bacteriocin | 1 | 9 |
| *Stenotrophomonas_*1 | NRPS | 2 | 55 |
| *Stenotrophomonas_*1 | LAP | 1 | 21 |
| *Stenotrophomonas_*1 | arylpolyene | 1 | 39 |
| *Acinetobacter_*19 | NRPS | 3 | 62 |
| *Acinetobacter_*19 | arylpolyene | 1 | 24 |
| *Acinetobacter_*19 | siderophore | 1 | 10 |
| *Acinetobacter_*19 | bacteriocin | 1 | 14 |
| *Acinetobacter_*19 | betalactone | 2 | 27 |
| *Acinetobacter_*19 | LAP, bacteriocin | 1 | 5 |
| *Acinetobacter_*19 | terpene | 1 | 16 |
| *Acinetobacter_*19 | hserlactone | 1 | 10 |

Table S4 Chitinase (EC: 3.2.1.14) of antagonistic strains.

| Sample ID | Chromosome location |
| --- | --- |
| *Bacillus_*1 | S6_GM000902 |
|  | S6_GM002583 |
| *Acinetobacter_*4 | S7_GM001709 |
| *Serratia_*1 | S9_GM004197 |
| *Bacillus_*2 | S10_GM003768 |
|  | S10_GM006506 |
| *Pseudomonas_*1 | S11_GM003217 |
|  | S11_GM007953 |
| *Stenotrophomonas_*1 | S29_GM000418 |
|  | S29_GM001064 |
| *Acinetobacter_*19 | S30_GM004226 |
|  | S30_GM004284 |

Table S5 Zwittermicin A and carotenoids of antagonistic strains.

| Sample ID | Chromosome location | Start | End | Most similar known cluster | Similarity |
| --- | --- | --- | --- | --- | --- |
| *Bacillus*_2 | Scaffold19 | 1 | 102613 | zwittermicin A | 100% |
| *Pantoea*_1 | Scaffold2 | 883471 | 907032 | carotenoid | 100% |

Fig S1 PCA analysis of biosynthetic gene clusters of antagonistic strains against *P. destructans* from bat skin and soil samples.

Fig S2 Comparative analysis of biosynthetic gene clusters in the *Bacillus*.

Isolates are labeled with genus and ID number. Dark red represents core biosynthetic genes; pink represents additional biosynthetic genes; blue represents transport-related genes; green represents regulatory genes; and gray represents other genes.

(A). Terpene (homology: 99%), (B). Bacteriocin (homology: 99.36%), (C). Siderophore (homology: 98.9%).
